# Protocol for the Effectiveness of an Anesthesiology Control Tower System in Improving Perioperative Quality Metrics and Clinical Outcomes: the TECTONICS randomized, pragmatic trial

**DOI:** 10.12688/f1000research.21016.1

**Published:** 2019-11-29

**Authors:** Christopher R. King, Joanna Abraham, Thomas G. Kannampallil, Bradley A. Fritz, Arbi Ben Abdallah, Yixin Chen, Bernadette Henrichs, Mary Politi, Brian A. Torres, Angela Mickle, Thaddeus P. Budelier, Sherry McKinnon, Stephen Gregory, Sachin Kheterpal, Troy Wildes, Michael S. Avidan, TECTONICS Research Group

**Affiliations:** 1 Department of Anesthesiology, Washington University in St Louis, St Louis, MO, 63110, USA; 2 Institute for Informatics, Washington University in St Louis, St Louis, MO, 63110, USA; 3 Department of Computer Science and Engineering, Washington University in St Louis, St Louis, MO, 63110, USA; 4 Department of Surgery, Washington University in St Louis, St Louis, MO, 63110, USA; 5 Department of Anesthesiology, University of Michigan, Ann Arbor, MI, 48109, USA

**Keywords:** Telemedicine, Artificial Intelligence, Machine Learning, Forecasting Algorithms, Decision Support, Randomized Controlled Trial, Anesthesiology

## Abstract

**Introduction: **Perioperative morbidity is a public health priority, and surgical volume is increasing rapidly. With advances in technology, there is an opportunity to research the utility of a telemedicine-based control center for anesthesia clinicians that assess risk, diagnoses negative patient trajectories, and implements evidence-based practices.

**Objectives:** The primary objective of this trial is to determine whether an anesthesiology control tower (ACT) prevents clinically relevant adverse postoperative outcomes including 30-day mortality, delirium, respiratory failure, and acute kidney injury. Secondary objectives are to determine whether the ACT improves perioperative quality of care metrics including management of temperature, mean arterial pressure, mean airway pressure with mechanical ventilation, blood glucose, anesthetic concentration, antibiotic redosing, and efficient fresh gas flow.

**Methods and analysis:** We are conducting a single center, randomized, controlled, phase 3 pragmatic clinical trial. A total of 58 operating rooms are randomized daily to receive support from the ACT or not. All adults (eighteen years and older) undergoing surgical procedures in these operating rooms are included and followed until 30 days after their surgery. Clinicians in operating rooms randomized to ACT support receive decision support from clinicians in the ACT. In operating rooms randomized to no intervention, the current standard of anesthesia care is delivered. The intention-to-treat principle will be followed for all analyses. Differences between groups will be presented with 99% confidence intervals; p-values <0.005 will be reported as providing compelling evidence, and p-values between 0.05 and 0.005 will be reported as providing suggestive evidence.

**Registration:** TECTONICS is registered on ClinicalTrials.gov, *NCT03923699*; registered on 23 April 2019.

## List of Abbreviations

ACT                 Anesthesiology Control Tower

ACTFAST        Anesthesiology Control Tower—Feedback Alerts to Supplement Treatments

AKI                  Acute Kidney Injury

BJH                  Barnes-Jewish Hospital

EHR                 Electronic Health Record

ML                   Machine Learning

NINR               National Institute of Nursing Research

PHI                  Protected Health Information

PI                     Principal Investigator

SMC                Safety Monitoring Committee

OR                   Operating Room

RCT                 Randomized Controlled Trial

TECTONICS   Telemedicine Control Tower for the OR: Navigating Information Care and Safety Trial

## Introduction

Perioperative complications collectively contribute to numerous deaths around the world ^1^. Following inpatient surgeries, the estimated 30-day mortality is between 1% and 5% ^2– 10^, and between 5% and 10% of surgical patients will die in the following year ^4, 5, 8, 11^. Furthermore, 10% to 20% of surgical patients experience major complications such as heart attacks, chronic pain ^12^, infections and blood clots following their procedures ^6, 9, 10, 13^. Although some complications are unavoidable, based on the nature of the particular surgical procedure or non-modifiable patient characteristics ^9, 14, 15^, others may be preventable through early identification of patient risk factors and the use of tailored treatments.

Several factors, besides technical aspects of surgeries, contribute towards preventable perioperative complications. Clinicians ^16– 19^, including anesthesia care teams (typically comprising anesthesiologists and certified registered nurse anesthetists [CRNAs]), sometimes fail to implement and adhere to evidence-based standards of care. Many clinicians believe that they are in compliance with guidelines, when in reality they are not ^20^. In the United States, differing backgrounds and perspectives of anesthesiologists and CRNAs can undermine effective collaboration among team members, potentially compromising patient care ^21, 22^. Clinicians also experience cognitive overload in the operating room (OR), and limitations in cognitive capacities impair information processing ^23^ and decision making abilities ^24– 26^. In addition, the rapidly evolving OR environment and complex patient responses to surgery and anesthesia make it challenging for clinicians to accurately assess patients’ shifting risks.

Telemedicine and integrated machine learning (ML) are two promising approaches for addressing cognitive and information overload, dynamically focusing resources where needed, and reducing accidental variations in care quality. However, there is no prospective evidence on telemedicine or ML in the OR context. There is an urgent need for rigorous research investigating the utility of a telemedicine-based control center to dynamically assess risk, diagnose negative patient trajectories, implement evidence-based practices, and improve outcomes for surgical patients. A collaborative telemedicine solution for the OR, through the early and accurate identification of potential risks, could facilitate the development of tailored plans for patient care risk mitigation and management ^27– 29^. It could also enhance meaningful teamwork between CRNAs and anesthesiologists, act as a complementary support for anesthesia care teams in the OR, help to decrease cognitive overload and bias, and facilitate evidence-based care.

To address this deficit, our interdisciplinary team, including academic and clinical leaders, has developed a prototype anesthesiology control tower (ACT) ^30, 31^. Our previous pilot work has demonstrated the usefulness and usability of the ACT ^30, 32^. We have also developed ML algorithms for real time decision-support instruments ^33– 36^, developed the institutional infrastructure to maximize OR integration of the ACT, and evaluated the feasibility of conducting a large scale randomized control trial using the ACT ^31^. In this protocol we outline the Telemedicine Control Tower for the OR: Navigating Information, Care and Safety (TECTONICS) trial, which is a large-scale randomized controlled trial (RCT) to empirically evaluate the effectiveness of the ACT on preventing clinically relevant adverse perioperative outcomes and improving perioperative care.

We hypothesize that the integrated ACT system in the TECTONICS trial will improve evidence-based quality of perioperative care metrics and prevent clinically relevant adverse perioperative outcomes (postoperative delirium, renal failure, respiratory failure, and 30-day mortality).

## Participants and setting

The study is a single center, randomized, controlled, phase 3, pragmatic superiority trial at Barnes Jewish Hospital (BJH) in St. Louis, MO. All adults (18 years and older) undergoing surgical procedures in these operating rooms will be included. Children are excluded in this study. Labor and operative delivery is conducted in a separate administrative area and is also excluded unless it occurs in the main surgical ORs. There are no other exclusion criteria related to procedure type, comorbid illnesses, or planned disposition other than the requirement that some anesthesia clinician be requested (excluding e.g. organ procurement and minor procedures performed without anesthesiology services) and the requirement for the procedure to take place in an operating room (excluding sedation-based procedure suites such as the cardiac diagnostic laboratory).

**Figure 1.**
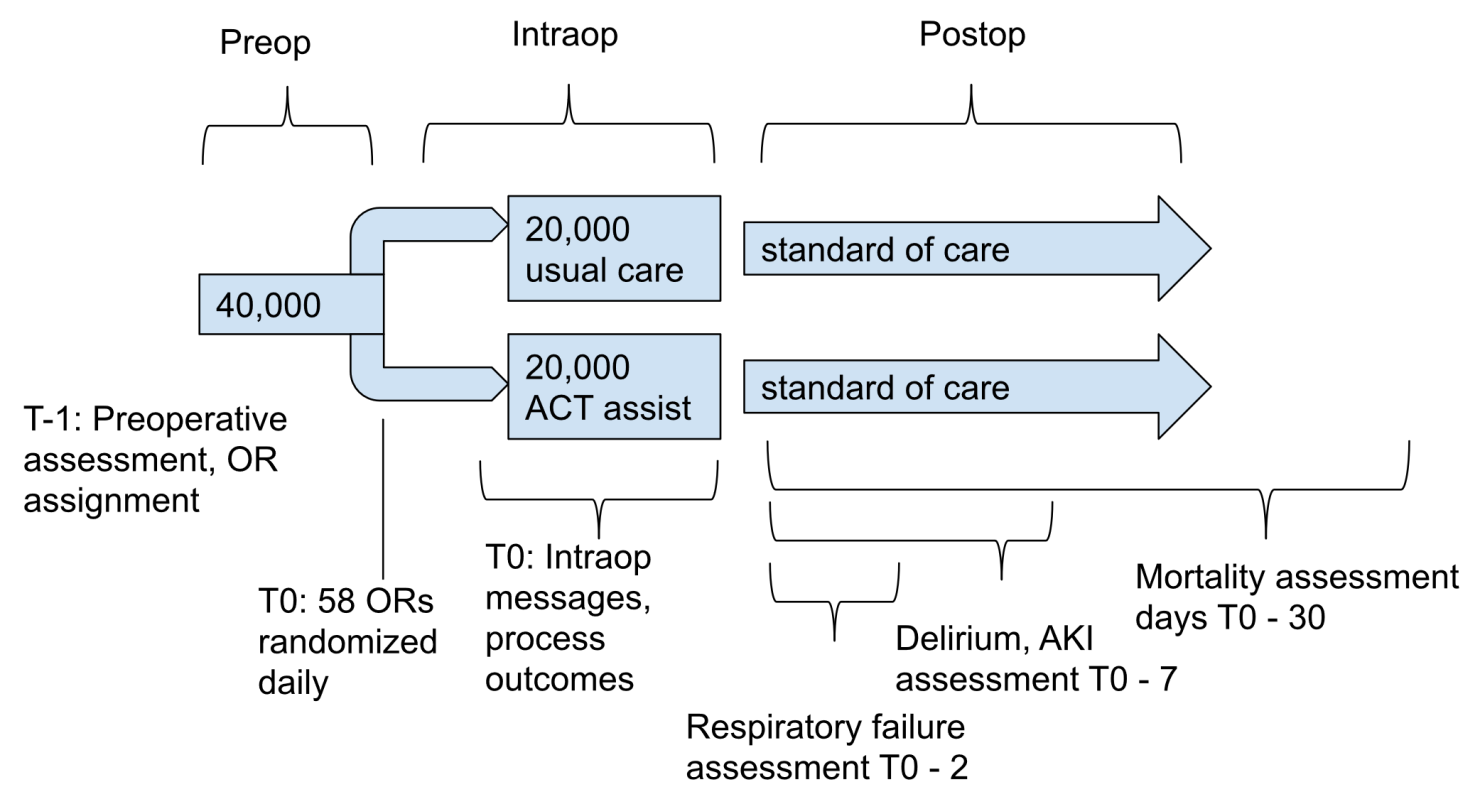
Schematic of study design, patient activity flow.

An estimated 10,000 patients will be enrolled annually, and enrollment will be over four years for approximately 40,000 total patients enrolled ( Figure 1). Cases started during the hours of operation of the ACT will be included regardless of the stop time.

This study has been approved by the Human Research Protection Office at Washington University (St. Louis, MO # 201903026) for enrollment with a waiver of consent. Participant data is collected from the electronic health record (EHR) of Barnes-Jewish and its affiliated hospital and clinic databases until 30-days after their surgery.

## Interventions

The participant groups are intraoperative telemedicine support from the ACT (“intervention”) and usual care.

### Description of the ACT

**Figure 2.**
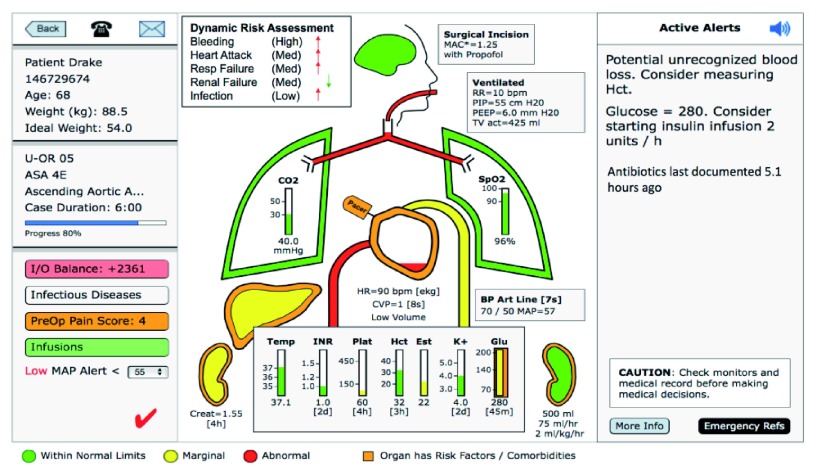
Summary overview data for a hypothetical patient (AlertWatch® ACT Dashboard).

**Figure 3.**
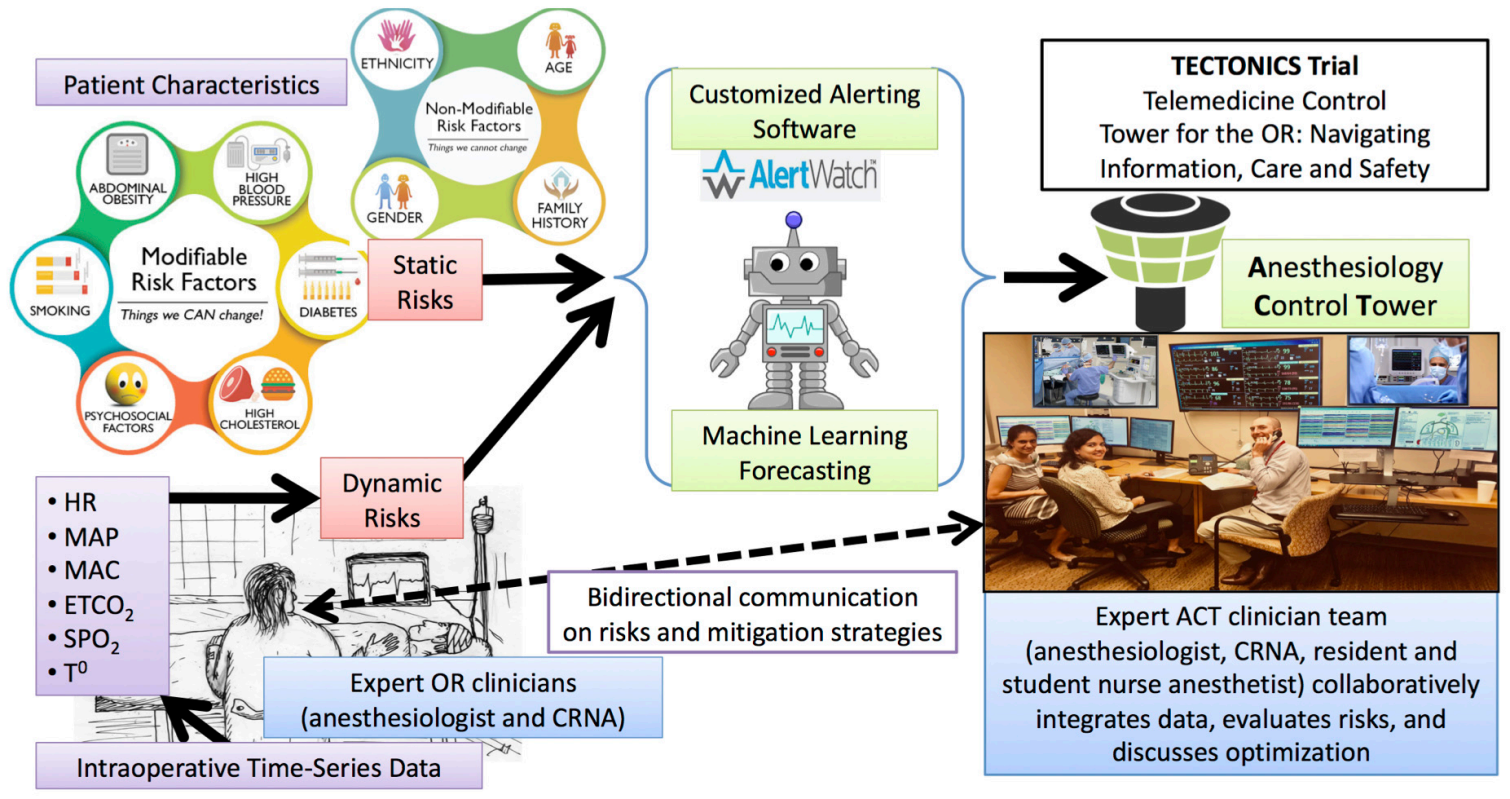
The key workflow and process components of TECTONICS. The team in the ACT receives data form the electronic health record, web-interfaced monitors in the operating room (OR), video cameras in the OR, multipath convolutional neural network machine learning algorithms, and alerting software has been customized to provide maximum utility in an ACT. The team weaves together disparate data strands, and collaboratively formulates a plan to address the patient’s risk and optimize outcomes. The plan is discussed collegially with OR clinicians, who exercise judgement in delivering the best individualized perioperative management to each surgical patient. Dynamic data from OR patient monitors. (The photo was taken in our prototype ACT). CRNA, certified registered nurse anesthetist.

The ACT has been established in a location remote from the OR with sophisticated hardware and software. It is staffed by at least two clinicians from the research team (an anesthesiologist and one or more other clinicians). Intraoperative data streams used by the ACT clinicians include real-time access to the hospital’s EHR, web-based visualization of vital signs and waveforms, treatment guidelines, protocols for care, as well as AlertWatch® (Ann Arbor, MI) ( Figure 2, Figure 3). AlertWatch® is a Food and Drug Administration-approved patient monitoring and alerting system that displays integrated patient information. AlertWatch® was primarily conceptualized for use in individual ORs; we have modified the AlertWatch® software based on stakeholder engagement and feedback for use in the ACT, creating a customized dashboard and interface specifically designed for the telemedicine setting ^32^. The ACT AlertWatch® version that we developed in our preliminary studies is a customized research product with innovative information technology and communication components and is distinct from the current commercial product in several important respects. Real-time forecasting (from the machine-learning algorithms discussed below) of adverse outcomes for individual patients is provided to ACT clinicians. BJH is currently installing high definition cameras in the ORs; video feeds will be incorporated in the ACT if these are available during the trial. Video or audio will not be stored.

### Intervention group

In ORs randomized to intervention, ACT clinicians contact OR clinicians in two phases. First, the ACT messages the OR clinician an individualized risk assessment and considerations / recommendations based on the preoperative evaluation and real time information from the monitors in the OR. The recommendations are geared towards (1) preventing major complications ( Table 1) based on patients’ specific risk profiles (e.g. history of stroke, hypertension, type 1 diabetes, coronary artery disease, valvular heart disease, pulmonary disease) and (2) adhering to general quality of care indicators ( Table 2). The recommendations are based on the best currently available evidence (e.g. intensive insulin management in type 1 diabetes). OR clinicians are encouraged to reply to the message with specific concerns they would like to discuss, risk assessments they believe to be erroneous, and additional monitoring that they would like the ACT to perform. The second phase occurs during procedures. The ACT monitors physiologic and process alerts generated by AlertWatch® and ML algorithms, filters these alerts for those believed relevant and actionable, and contacts the anesthesia team where deemed appropriate. OR clinicians receiving notification from the ACT may choose to carry out whatever course of action they deem clinically appropriate. Anesthesia clinicians in the OR have access to the institution’s “clinical” AlertWatch software, but do not have access to the “research” view.

**Table 1.** Primary outcome measures and definitions.

Measurement	Definition
Thirty-day postoperative mortality	Definition postoperative mortality provided by Johnson *et al*. This will include death of any cause occurring in or out of the hospital, within 30 days of the index surgery ^37^.
Postoperative delirium	Defined as an acute change in consciousness or cognition. It has a fluctuating course, and is characterized by inattention, disorganized thinking and altered level of consciousness. We have trained the nursing staff on our surgical intensive care units to assess all patients for postoperative delirium using the Confusion Assessment Method for the Intensive Care Unit (CAM-ICU) instrument ^38^. It is administered every 12–24 hours depending on clinical context while in the ICU. All delirium assessments within 7 days will be included.
Postoperative respiratory failure	Defined as mechanical ventilation for greater than 24 hours after surgery, or unplanned postoperative re- intubation and mechanical ventilation within 48 hours of surgery ^39^. Planned staged operations are excluded.
Postoperative acute kidney injury	Diagnosed when any of the following three criteria are met: (i) an increase in serum creatinine by 50% compared with preoperative within 7 days, (ii) any increase in serum creatinine > 0.3 mg/dL in 48 hours, or (iii) oliguria (urine output <0.5 mL/kg/hr for 6–12 hours) ^33, 40, 41^.

**Table 2.** Secondary outcome measures and definitions.

Measurement	Definition
Temperature management	Temperature ≥ 36°C at end of surgery
Antibiotic redosing	Antibiotic redosing compliant with guidelines developed by the institutional pharmacy and therapeutics committee.
Mean arterial pressure management	Percentage time during surgery with mean arterial pressure ≥ 60 mmHg
Mean airway pressure with mechanical ventilation	Percentage time during surgery with mean airway pressure ≤ 30 cmH _2_O.
Blood glucose management	Proportion of patients with blood glucose ≤ 200 mg/dL at end of surgery.
Measured anesthetic concentration	Proportion of patients without ≥ 15 consecutive min of anesthetic concentration ≤ 0.3 MAC during anesthetic maintenance period.
Fresh gas flow rates	Proportion of patients with efficient fresh gas flow for ≥90% of the anesthetic maintenance period

### Usual care

In ORs randomized to usual care, the ACT monitors patients, but the ACT clinicians does not contact the OR clinicians unless the ACT clinicians believe it to be clinically necessary for patient safety purposes (e.g. neuromuscular blockade without evidence of hypnotic agent administered). Concomitant care is provided in the usual perioperative setting with no modifications based on the trial. Anesthesia clinicians in the OR have access to the institution’s “clinical” AlertWatch software, but do not have access to the “research” view.

### Risk forecasting algorithms

Using data from over 110,000 patients, we developed calibrated ML algorithms to predict adverse postoperative outcomes. The details of the dataset and algorithm development are published elsewhere ^34– 36^. Briefly, we implemented deep neural network models for 30-day mortality, acute kidney injury, and postoperative ventilatory failure among other anesthesiology-relevant outcomes based on routinely collected clinical data. We tested numerous other prediction methods, and found that deep neural networks processing extensive patient information (i.e., demographic characteristics, surgical risk, co-morbidities) as well as time series physiological data (e.g., blood pressure, temperature, heart rate) predict outcomes such as death with high area under receiver operator characteristics curve (0.880), acceptable sensitivity (~50%) and excellent specificity (~95%). We adapted models to improve their interpretability and incorporated advanced post-processing methods to uncover the data which drives individual predictions. The training of these deep neural network models jointly estimates data filtering and imputation steps with prediction ^33^. We implemented appropriate data validation and quality filtering steps for the live environment and display forecasts of mortality updated every 5 minutes. In contrast to standard forecasting models, we have previously demonstrated that ML and data mining approaches for patients in ICUs are markedly superior in predicting clinical outcomes such as mortality ^42^. The feasibility of this integration is supported by a previous successful trial, where members of our investigative team, using live data from the EHR, implemented ML algorithms to guide a rapid response team in medical wards ^43– 46^.

Over the course of the TECTONICS study, with ongoing acquisition of high-resolution data and outcomes on thousands of surgical patients, our algorithms will undergo regular evaluation and refinement. Periodically updating the model will be necessary, which we plan to do at 6-month intervals with newly collected data in the control arm. We will also use a human expert to review the face validity of the model’s predictions and most important input features. Such a dynamic feedback loop will continuously improve and adjust the model. We will test for the overall percentage of correct forecasts, the percent of correctly forecasted events, and accuracy when data include noise and missing values on several adverse perioperative outcomes. One key metric that we will evaluate and compare will be the sensitivity at 95% specificity, since it is important to maintain a high specificity (i.e., low false alarm rate) for meaningful decision support. Validation techniques will include cross-validation ^47^ and systematically different hold-out samples (e.g. distinct time periods or OR locations).

## Outcome measures and data acquisition

The primary objective is to determine whether the ACT system is effective in preventing clinically relevant adverse perioperative outcomes including thirty-day postoperative mortality, postoperative delirium, postoperative respiratory failure and postoperative acute kidney injury. Secondary objectives are to determine whether the ACT system is effective at improving perioperative quality of care metrics. The study outcomes will be assessed according to established criteria ( Table 1 and Table 2).

Multiple sources are used for standardized data collection. Data on patient outcomes and perioperative care metrics is extracted from the EHR. Preoperative patient characteristics, comorbidities, surgical and clinical history, as well as perioperative and immediate post-operative information are pulled from the EPIC EHR (Verona, WI, USA). Additional postoperative patient outcomes data (for sub-studies) will be obtained from clinical registries (American College of Surgeons National Surgical Quality Improvement Program ^48^, Society of Thoracic Surgeons ^49^,) as well as the EHR. BJH determines vital status through multiple mechanisms including follow-up contact and state death databases. Non-mortality outcomes are not tracked after discharge from hospital. Although the development of incident serious acute kidney injury (AKI) or delirium post-discharge is possible, we anticipate that these will be uncommon enough to not warrant large-scale surveillance.

Data on clinician responses to individual alerts, generated from the AlertWatch® Control Tower platform, is logged by ACT staff to a database for qualitative studies and internal quality improvement.

## Assignment of treatments

The 58 ORs (all the ORs at BJH excluding “remote” locations, procedure suites, and labor and delivery) are 1:1 randomized daily to receive intraoperative support from the ACT or usual care without any form of stratification. In other words, participants are randomized in clusters whose size randomly depends on the number of cases assigned to a room. The randomization script for the TECTONICS trial has been incorporated into the AlertWatch® ACT infrastructure, and runs automatically early every morning.

Due to the nature of the intervention it is not possible to blind OR clinicians since feedback alerts from the ACT inform them that they are in the intervention group. However, patients and those evaluating outcomes are blinded to group assignment.

## Sample size calculation and recruitment

In recent years, more than 20,000 surgeries have been performed annually in the Barnes Jewish Hospital main ORs. However, the ACT is only staffed during work-week hours and when the assigned attending anesthesiologist is available. During our pilot, we made efforts to ensure staffing during all appropriate times; however, under half of cases took place during ACT staffed times. We therefore conservatively estimate that 10,000 patients will participate per year, but we anticipate that more rapid accrual is likely based on more complete recent staffing. The TECTONICS trial will be adequately powered to answer with precision whether the ACT system has a meaningful impact on clinically relevant outcomes (primary outcomes, see Table 3) and quality of care metrics (secondary outcomes, see Table 4). Despite the fact that we are evaluating multiple surrogate outcomes, the very large sample size will provide adequate statistical power to determine whether or not there is improvement with the ACT system. Individuals with multiple surgeries within 30 days will be analyzed with the assignment of their index surgery. Individuals with multiple independent encounters (>30 days separation) will be treated as distinct observations. Surgeries which take place outside BJH will not be accounted for.

**Table 3.** Primary outcomes to be assessed with estimation of power for each metric.

Primary adverse outcomes	Estimated current incidence	Target with ACT support	Power based on 40,000 patients in RCT (p<0.005)
Thirty-day postoperative mortality	2% ^2- 5^	1.5%	84% (>80%) *
Postoperative delirium (only patients admitted to intensive care units [ICUs])	25% ^50, 51^	21%	93%% (>80%) * (Based on 8,000 patients admitted to ICU)
Postoperative respiratory failure	2% ^52^	1.5%	84% (>80%) *
Postoperative acute kidney injury	2% ^53, 54^	1.5%	84% (>80%) *

*The adjusted power was calculated assuming a cluster-randomized design allowing for an intracluster correlation between 0.005 and 0.01 and varying number of patients per OR. The current incidence estimates on which these power analyses are based are consistent with findings in our previous studies ^4, 5, 12, 55, 56, 57, 58, 50, 51^ as well as from the ACTFAST _2_ pilot study, where we have approximations of these complications from ~110,000 (mostly inpatient) surgical patients at our institution over 5 years.

**Table 4.** Secondary outcomes to be assessed with estimation of power for each metric.

Secondary outcome measures	Estimated current compliance	Target with ACT support	Power based on 40,000 patients in RCT (p<0.005)
Temperature ≥ 36°C at end of surgery	60%	70%	>99% (>90%) *
Antibiotic redosing adherence ≥ 90%	70%	90%	>99% (>90%) *
Percentage time during surgery with mean arterial pressure ≥ 60 mmHg	80%	85%	>99% (>90%) *
Percentage time with peak airway pressure ≤ 30 cmH _2_O	75%	85%	>99% (>90%) *
Proportion with blood glucose ≤ 200 mg/dL at end of surgery	75%	85%	>99% (>90%) *
Proportion without ≥ 15 consecutive min of anesthetic concentration ≤ 0.3 MAC during maintenance period	95%	99%	>99% (>90%) *
Proportion with efficient fresh gas flow for ≥90% of anesthetic period	75%	90%	>99% (>90%) *

*The adjusted power was calculated assuming a cluster-randomized design allowing for an intracluster correlation between 0.01 and 0.03 and varying number of patients per OR for a total N=40,000.

## Assessing Hawthorne and contamination effects

We anticipate a possible contamination (or learning) effect over time in the usual care group. Clinicians are included in both intervention and control ORs (possibly on the same day) and may become sensitized to the standards of practice and the surrogate outcome measures being tracked, leading to “overlapping” improvements in these measures (as well as clinical outcomes) in both groups over the course of the study. This learning effect might manifest most strongly among clinicians who spend time in the ACT. Furthermore, with the knowledge that clinical behaviors are being observed, there is a high likelihood of a Hawthorne effect ^59, 60^. In the reverse of the usual Hawthorne problem, the effect of being observed *in the intervention arm* represents part of the actual effect of the intervention; knowing that they are being observed, clinicians may feel more accountable to following perioperative best practices, and this is a meaningful effect. However, the non-contact rooms are also aware of the trial and enjoy the same improvement, falsely decreasing the estimated effect size. The data we have obtained prior to instituting the ACT will be useful in assessing the extent of contamination. Specifically, we will assess the intensity/frequency of contamination by comparing the outcomes of the control group patients to those of matched patients who had similar surgeries, demographics, and health conditions during the immediate period prior to the ACT implementation. We will also analyze control arm results in 3-month time bins (with baseline data for reference) to evaluate secular tends which may represent contamination, as well as the magnitude of clustering design effects.

## Analysis of primary and secondary outcomes

Comparisons between groups during the randomized study will be with parametric and non-parametric statistical tests, according to the distributions of the measures of interest. Fisher’s exact or χ ^2^ test will be used to assess differences between proportions (the majority of assessments). Contingency statistical tests will be used to compare occurrence of hypotension, hyperglycemia, hypoglycemia, and hypothermia between groups; and unpaired t-test or Mann-Whitney U-test, as appropriate, will be used to compare the durations of these occurrences between groups. All comparisons will use the intention-to-treat principle.

Results in statistical tests with a p value <0.005 will be viewed as providing compelling evidence, whereas results with a p value between 0.05 and 0.005 will be interpreted as providing suggestive evidence ^61^. We will report p-values adjusted for multiple hypothesis tests (within the primary and secondary outcome blocks) using permutation methods that account for the correlation across outcomes ^62^. Within the secondary outcomes, false-discovery-rate control will be reported ^63^.

## Handling of missing data

We anticipate that the prospectively collected data will be high quality with few missing outcomes. AKI is informatively missing in patients who are judged as low risk by the surgical team and do not have assessments of postoperative creatinine or urine output. Delirium is similarly infrequently assessed at our institution in patients who do not require intensive care unit admission. A screening bias in both outcomes is possible where patients in the treatment arm are more accurately identified as elevated risk and checked for complications. Ventilatory failure is unlikely to occur in the 48-hour time window among discharged patents. We will report the number of patients without assessments for each outcome. The primary analysis will treat patients discharged without measures of AKI or delirium as negative. Patients who are informatively censored by death will be treated as positive for other outcomes. All outcomes will be required to be incident. Individuals without preoperative measures of renal status or delirium will be assumed to have normal values.

## Adverse event and safety monitoring

This study will have a Safety Monitoring Committee (SMC) according to the National Institute of Nursing (NINR) Research Data Safety Monitoring policy. The SMC will be comprised of a small group of experts with at least two members independent of the study team. They will be responsible for reviewing all adverse events, compliance with the IRB requirements, investigator compliance, minimizing risks and protecting the confidentiality of participant data. The SMC will review study data every six months.

The study team will prepare reports for the SMC, NINR and the IRB. In the event a serious adverse event occurs that is deemed to be reasonably associated with the conduct of the study by an external SMC, the study may be halted. Local regulatory agents in agreement with the study principle investigator may decide to stop the study at any time.

## Strengths, limitations and alternative strategies

The TECTONICS trial has important strengths. It is a pragmatic RCT conducted in a high volume, real-world clinical setting that incorporates telemedicine for the OR. The adverse outcomes under study are serious and meaningful to patients. The TECTONICS trial can be conducted efficiently as many components of the proposed study are incorporated into existing infrastructures and processes at Washington University: 1) with no known risk associated with the support offered by the ACT, participants are included with a waiver of informed consent; 2) the members of the care team (anesthesiologists, CRNAs, residents and student registered nurse anesthetists) participate in the trial in the course of their routine clinical work; and 3) most of the surrogate and clinical outcomes data are obtained from existing IT resources or from established and ongoing registries ^64^. Randomization of ORs is implemented easily, and the process for providing feedback alerts from the ACT does not require any lead-in time or advanced preparation. The study includes all adult surgical-patients at BJH, including both men and women, and those who are recognized to be vulnerable and understudied in clinical research. The feasibility of the trial is enhanced by participation of a highly committed cadre of CRNAs and attending anesthesiologists, student registered nurse anesthetists and residents in the Anesthesiology Department, as well as an experienced team of CRNA and anesthesiology investigators that has established a track record of scientific collaboration and completion of major trials ^4, 5, 55– 57, 65– 67^.

There are also relevant limitations. The TECTONICS study will be vulnerable both to Hawthorne and contamination effects. Although we do not think that these effects can be eliminated, we have considered how best to account for them in the analyses. In addition, it will not be possible to ensure blinding ^68^ of clinicians. However, surgical patients and those evaluating outcomes will be blinded to group assignment. Another major constraint relates to both accuracy and completeness of outcome measures. Outcomes routinely tracked in the EHR are often well represented. However, we know from previous experience that EHR, registry, and patient reported outcomes data are occasionally inaccurate ^58^. Missing and inaccurate outcomes data will be partially mitigated by the large number of patients included in the trial, and are expected to be randomly distributed across groups.

## Ethics/protection of human subjects

This study has been approved by the Human Research Protection Office at Washington University (St. Louis, MO # 201903026). It satisfies the criteria for a waiver of informed consent (there is minimal risk with the intervention, the research could not practically be conducted without a waiver, and the rights and welfare of patients are not adversely affected by their involvement in the study, and there is no deception requiring additional disclosure) and is being conducted accordingly. This protocol was written in compliance with the SPIRIT Checklist Guidelines for Interventional Trials. Only the minimum necessary private patient information will be collected for the purposes of the study. Any protected health data is kept in a secure digital environment that is digitally encrypted, password protected and limited to research team only. De-identified data may be kept and used in future studies not pre-specified in the above protocol. The investigators are responsible for ensuring the accuracy, completeness, legibility, and timeliness of the data collected.

## Data management

The ACT will use clinical applications available to all clinicians to monitor ongoing surgical procedures. These applications can only be accessed over the secure hospital network or by virtual private network logins. This arrangement meets and/or exceeds Health Insurance Portability and Accountability Act standards for Patient Health Information (PHI) security. AlertWatch® is an approved clinical application at Barnes-Jewish Hospital, and therefore maintains these same levels of protection. Access to the data collected in this study will be restricted to approved personnel. It is a strict policy that PHI content cannot be reviewed outside of this protected environment. Prior to data analysis, information will be de-identified. When possible, extracts from this project will avoid the use of PHI. De-identified patients can be identified using a special, non-PHI primary key, which we have used previously.

The research material obtained in this proposed trial will consist of the already established infrastructure and resources of the SATISFY-SOS (NCT02032030), NSQIP and STS registry studies, Anesthesiology Control Tower—Feedback Alerts to Supplement Treatments (ACTFAST) 1, 2 and 3 studies, the intra-operative electronic medical record and the AlertWatch® evidence-based alerting system. Patient demographic information and preoperative characteristics are collected and entered into the electronic health record as part of routine clinical care at the Center for Preoperative Assessment and Planning. Perioperative and intraoperative drug administration and vital signs are also charted in the electronic health record.

Data will be maintained for at least 5 years after the end of the grant funding period. Further study record retention will be at the discretion of the study investigator. Data may be used for future studies not mentioned in the protocol.

## Publication/data sharing policy

The investigative team is comprised of a range of stakeholders, including scientists, clinical investigators, and relevant end users. We will each disseminate in our respective networks through presentations to relevant stakeholder groups, through peer-reviewed publications, and by providing brief summaries for hospital administrators and policy makers. We will also utilize the BJC Collaborative, which aligns multiple health networks in the region (including rural settings), as a vehicle for dissemination.

Data from the TECTONICS trial will be made available for analysis in compliance with the recommendations of the International Committee of Medical Journal Editors ^69^. For this study, individual participant data that underlie the results of the trial will be made available after appropriate de-identification, along with the study protocol and statistical analysis plan. We plan to make this information accessible to researchers who provide a methodologically appropriate proposal for the purpose of achieving the aims of that proposal. Data will be available beginning 9 months and ending 36 months following trial publication at a third-party website. Data requestors will need to sign a data access agreement to gain access to trial data. Proposals should be directed to avidanm@wustl.edu. TECTONICS is registered on clinicaltrials.gov, NCT03923699.

## Authorship eligibility and contributorship

Authorship for this study will be given to key personnel involved in study design, data collection, and data analysis. There are no publication restrictions and no professional writers will be involved in the generation of the manuscript. M. Avidan, A. Ben Abdallah, J. Abraham, Y. Chen, B. Fritz, C. King, B. Henrichs, T. Kannampallil, M. Politi, A. Sharma, B. Torres, S. Kheterpal, T. Wildes are responsible for conceptualizing study design.

All authors, including King, Avidan, Ben Abdallah, Abraham, Chen, Fritz, Henrichs, Kannampallil, Politi, Sharma, Torres, Mickle, Budelier, McKinnon, Gregory, Wildes, have critically revised the TECTONICS protocol and approved the final version. All authors agree to be accountable for the accuracy and integrity of all aspects of the TECTONICS trial. No paid writers will be used.

## Protocol amendments

Protocol amendments will be approved by the steering, operations, safety, and data management committees, communicated to the IRB and NINR, and posted to Clinicaltrials.gov. This protocol corresponds to version 1.1 and was agreed on October 1 2019.

## Conclusion

While ML and telemedicine have been extensively studied over the past decade, telemedicine has often been implemented without a strong research foundation. When it has been studied, this has often been done in the context of observational before and after cohort trials. ML algorithms have often been studied for risk calculations and predictions, but there has been limited investigation of their application to improving patient outcomes. In contrast, TECTONICS is designed as a pragmatic, randomized clinical trial including telemedicine and ML. The over-arching strength of TECTONICS is that it combines and leverages a telemedicine initiative with advanced machine-learning algorithms. The net innovation is a fully integrated clinical decision support system, comprised of remote surveillance of patient risks in real-time, human expert judgment, and computer-generated rules. This realization of the ACT concept provides an empowering and unobtrusive socio-technical telemedicine infrastructure and decision support solution for OR teams. The ACT also provides a practical and innovative solution to the challenge of implementing evidence-based guidelines in the OR. Although the ACT system requires an initial modest financial investment, if it proves to be effective in promoting and enhancing evidence-based perioperative care, it is possible that it could lead to decreased costs via improvements in surgical patient outcomes.

The proposed study can have a major impact on healthcare if it demonstrates that the ACT system enhances OR care quality and patient safety while simultaneously increasing teamwork. Following the TECTONICS study, the ACT system will be further refined, and its implementation will be expanded. The logical next step will be to conduct a larger multisite trial focusing on expanded clinical and patient-reported outcomes. We are well positioned to track such outcomes, based on electronic access to ICD codes ^58^, our experience in building a patient reported outcomes registry ^50, 58, 70^, as well as our collaborations with NSQIP and the Society for Thoracic Surgeons. Importantly, one of the TECTONICS contributors (Kheterpal) is the principal investigator of the Multi-Center Perioperative Outcomes Group (MPOG), which includes data from >4 million patients, and has an established and sophisticated international IT infrastructure. We envisage that future dissemination and implementation of the ACT system could occur efficiently using the MPOG infrastructure.

Throughout the study, we will collect data from ACT users on reach (percent of clinicians and staff eligible who use and engage with the ACT), adoption (user confidence that they will continue to use the ACT after the study ends), implementation (user confidence that the ACT can be consistently delivered as intended), and maintenance (user confidence that the ACT will produce lasting benefits beyond the study) ^71^. When planning for wider-scale implementation, we will use established guidelines set-out by the Expert Recommendations in Implementing Change (ERIC) team ^72^.

## Data availability

### Underlying data

No data are associated with this article.

### Reporting guidelines

Repository: SPIRIT checklist for ‘Protocol for the Effectiveness of an Anesthesiology Control Tower System in Improving Perioperative Quality Metrics and Clinical Outcomes: the TECTONICS randomized, pragmatic trial’. https://doi.org/10.6084/m9.figshare.10255724 ^73^.

Data are available under the terms of the Creative Commons Attribution 4.0 International license (CC-BY 4.0).
