# Supplementary material for: Protocol for the Effectiveness of an Anesthesiology Control Tower System in Improving Perioperative Quality Metrics and Clinical Outcomes: the TECTONICS randomized, pragmatic trial
Source: F1000Res. 2019 Nov 29;8:2032. [Version 1] doi: 10.12688/f1000research.21016.1 (PMC7076336; doi:10.12688/f1000research.21016.1)
Supplement: Peer review history

# Peer review: Reviewer response for version 1

**DOI:** 10.5256/f1000research.23127.r60684

**Published:** 2020-03-16

**Authors:** Leon du Toit, Ross Hofmeyr, Maretha Smit

**Affiliations:** 1 Department of Anaesthesia and Perioperative Medicine, Groote Schuur Hospital, University of Cape Town, Cape Town, South Africa; 2 Department of Anaesthesia and Perioperative Medicine, Groote Schuur Hospital, University of Cape Town, Cape Town, South Africa

This protocol originates from a cross-disciplinary group of researchers including perioperative clinicians and data scientists. The project is based at a single academic hospital in North America. The protocol describes a pragmatic clinical trial of a complex systems intervention using machine learning guided remote decision support in anaesthesia care. The primary outcome is a composite of 30-day mortality, postoperative delirium, respiratory failure and acute kidney injury after surgery. The proposed trial is pragmatic in design.

1. Population: The population includes all adult surgical patients taking place in the main theatre facilities of the trial site. Obstetric patients and other patients undergoing surgery in locations remote to the main theatre facilities are excluded by the sampling method. The study population is restricted to a single academic hospital in a high-income setting.
2. Intervention: The intervention is complex in design and implementation, but all the components of the intervention have already been piloted and introduced into the system and the technology supporting the trial are not new to the trial site. It is stated that the intervention will change over the duration of the trial as guidelines and updated and machine learning algorithms improve. Will the effect of an evolving intervention be evaluated? The complexity of the intervention should be considered when interpreting the findings of this trial.
3. Outcome: The outcome is clinically important to patients and healthcare providers. The components of the composite outcome are measured as part of routine care (data extracted from electronic medical records). The mortality component of the composite outcome is robust, but the event rate of the other components will likely be underestimated by this method of data collection. This will not affect validity of the estimate of effect of the intervention. The reviewers wonder what reasoning determined the components of the composite outcome. Why, for example, are cardiovascular events and surgical site infections not included?
4. Design: The unit of randomisation is the operating room, and randomisation is updated daily. Theoretically, randomisation to a complex intervention within a single institution risks contamination across the arms of the study. The investigators address this concern appropriately in the protocol. The concern of bias introduced by the Hawthorne effect is also addressed in the analysis plan. With a projected sample size of 40000 surgical cases, the proposed trial is sufficiently powered to answer the patient relevant question.
5. Burden: Despite the complexity of the trial, it does not pose additional burden to healthcare providers. Data collection is part of routine care, provision of the intervention does not rely on the usual healthcare providers, and the institutional review board approved a waiver of consent.

The reviewers wonder which ethical considerations were discussed for observing healthcare providers. Is there an agreement for participation process for clinicians who are being observed in this study?

 What will be considered an adverse event in this trial? Can examples be given? Will the project use specific criteria to identify adverse events?

 Monitoring and evaluation of the program would add value. What is the explicit logical framework connecting the intervention to the outcomes being measured? When reading the result of this trial the reviewers would conceivably be interested in knowing how the intervention lead to a change in management? What factors determined translation of recommendation from the ACT to practice change in theatre? Does the person sitting in the ACT cause variability in the way the intervention is delivered and received? Will the trial measure compliance or adherence to the ACT recommended intervention?  

 The capital investment and running cost of this intervention must be significant. Future work, investigating the cost of benefit will facilitate considerations around generalisability of this intervention.

 This protocol represents an exciting frontier in the field of anaesthesia research. The proposed trial incorporates precision care into routine care, assessed in a pragmatic manner. The reviewers look forward to the results and report of this trial, and future projects that build on this one.

We confirm that we have read this submission and believe that we have an appropriate level of expertise to confirm that it is of an acceptable scientific standard.

# Peer review: Reviewer response for version 1

**DOI:** 10.5256/f1000research.23127.r58762

**Published:** 2020-01-20

**Authors:** Ben Gibbison

**Affiliations:** 1 Department of Anaesthesia, Bristol Medical School, University of Bristol, Bristol, UK

This manuscript is a protocol and therefore this review affects that. 

 The authors plan to implement a combined ML based patient parameter monitoring / alert system and human advisory system to aid in decision support. Operating rooms are individually randomised by day to the intervention or simply monitoring of the physiological parameters ("control" group). This will take place in a large University Hospital system in the USA.

 Outcomes assessed include: thirty-day postoperative mortality, postoperative delirium, postoperative respiratory failure and postoperative acute kidney injury.

 The manuscript is well written and will be a fascinating study if and when they complete it.

I confirm that I have read this submission and believe that I have an appropriate level of expertise to confirm that it is of an acceptable scientific standard.
